# Comparative Study of Fluorescence Emission of Fisetin, Luteolin and Quercetin Powders and Solutions: Further Evidence of the ESIPT Process

**DOI:** 10.3390/bios14090413

**Published:** 2024-08-26

**Authors:** Alexandra Deriabina, Tatiana Prutskij, Hector Daniel Morales Ochoa, Esteban Delgado Curiel, Veranda Palacios Corte

**Affiliations:** 1 Faculty of Physical and Mathematical Sciences, Autonomous University of Puebla (BUAP), Puebla 72570, Mexico; alexandra.deriabina@correo.buap.mx (A.D.);; 2 Sciences Institute, Autonomous University of Puebla (BUAP), Puebla 72570, Mexico

**Keywords:** fisetin, quercetin, luteolin, fluorescence, TDDFT, ESIPT, system of notation for flavonoids

## Abstract

Fisetin and Luteolin are important flavonoids produced in plants and known for their antioxidant, anti-inflammatory, neuroprotective, and analgesic properties. They are also good candidates for different types of biosensors. The model used to describe the fluorescence (FL) emission of these flavonoids involves an excited-state intermolecular proton transfer (ESIPT) process that causes a change in the molecule configuration and a corresponding decrease in the emission energy. Due to the different molecular structures of Fisetin and Luteolin, only one possible proton transfer within the molecule is allowed for each of them: transfer of the H3 proton for Fisetin and of the H5 for Luteolin. Here, we compare their calculated emission wavelengths, obtained using TDDFT/M06-2X/6-31++G(d,p), with their FL emission spectra measured on the corresponding powders and solutions and show that the experimental data are consistent with the presence of the ESIPT process. We also compare the emission wavelengths found for Fisetin and Luteolin with those calculated and measured for Quercetin, where, under photoexcitation, the transfers of both H3 and H5 protons are possible. We analyze the difference in the processes associated with the H3 and H5 proton transfers and discuss the reason for the predominance of the H5 proton transfer in Quercetin. Additionally, a new system of notation for flavonoid molecules is developed.

## 1. Introduction

Fisetin (F) and Luteolin (L) are important flavonoids found in several plants and known for their ability to protect the human body from the damage caused by oxidative stress reactions associated with the pathogenesis of many chronic health problems such as neurodegenerative diseases, emphysema, and cardiovascular diseases, among others [1,2]. Various studies have shown that flavonoids are involved in antioxidant mechanisms, including direct scavenging of reactive oxygen species, activation of antioxidant enzymes, and metal chelating activity [3]. In addition, the unique photophysical properties of flavonoids make them promising for use in various types of biosensors based on fluorescent probes for sensing anions, metal ions, and small and macro biomolecules [4,5,6,7,8].

Theoretical and experimental studies of their optical properties are important for their possible application and for gaining better fundamental knowledge of the relation between their molecular structure and its characteristic optical wavelengths. Furthermore, comparison of the calculated wavelength values with those obtained from measurement will lead to further understanding of the nature of the radiation process.

All earlier experimental studies of the absorption and emission spectra of L and F were carried out on solutions with very low concentrations. For F, measurements of the absorption wavelength in methanol [9,10], methanol-water [11] and Tris-HCL buffer [12] solutions with concentrations ranging from 5 to 25 μM consistently showed a wavelength value of 362 nm. The maxima of the fluorescence (FL) spectra, measured on the same solutions using the excitation wavelength of 370 nm, were found at approximately 480 nm for solutions in methanol and in Tris-HCL buffer, and at 470 nm for solutions in methanol-water. The absorption wavelength of L solutions in methanol was found at 349 nm [9,13] and at 402 nm for solutions in alkaline methanol-water [14]. The FL emission intensity of L solutions has been reported to be very small [14,15]; nonetheless, the FL maxima were found at approximately 420 nm in methanol solution when an excitation wavelength of 355 nm was used [13], and at 520 nm in alkaline methanol-water solution when using an excitation wavelength of 384 nm [14].

To calculate the characteristic absorption and emission wavelengths of different organic molecules, the excited-state intermolecular proton transfer (ESIPT) is often considered. The ESIPT process consists of the transfer of a proton, upon photoexcitation, from the hydrogen bond donor group to its adjacent acceptor group, thus inducing a change in the molecule tautomeric form and a subsequent decrease in the radiative transition energy [16]. Since 1955, when the ESIPT process was for the first time suggested to explain the FL spectra of salicylic acid [17,18], it was extensively used for explaining and predicting the fluorescence spectra of many organic molecules, such as derivatives of pyrazolines [19], benzazoles [20], phenothiazines [21], anthraquinones [22], and several dyes [23,24,25], among others. The ESIPT process was also successfully taken into account in the studies of emission properties of several flavonoid molecules, for example, of Quercetin (Q) [26,27,28,29,30], Morin [31], Kaempferol [29,30,32] and Myricetin [30,33].

Characteristic wavelengths of absorption and emission of F and L molecules have been calculated using density functional theory (DFT) with different functionals. The calculated absorption wavelength (**λ_ab_**) of 354 nm and emission wavelength (**λ_em_**) of 486 nm corresponding to the enol form were found using the PBE0 functional [11]. Using PBE0/COSMO, the values for **λ_ab_** of 364 nm and **λ_em_** of 473 nm were obtained. In Ref. [34] using the B3LYP/PCM model and acetonitrile as a solvent, the values of a **λ_ab_** (**λ_em_**) of 368 nm (434 nm) for the enol form and **λ_ab_** (**λ_em_**) of 487 nm (550 nm) for the keto O3 form of the F molecule were found. For the L molecule, only the results obtained using the B3LYP functional are available. In Ref. [35], using the B3LYP/CPCM model and aqueous solvent, the **λ_ab_** of 358 nm, and **λ_em_** of 454 nm for the enol form and of 670 nm for the keto O5 form of the L molecule were found. In calculations carried out in Ref. [13], the B3LYP-D3/PCM method and methanol as a solvent were considered, obtaining the values of 352 nm for **λ_ab_** and 417 nm for **λ_em_**.

**Figure 1:**
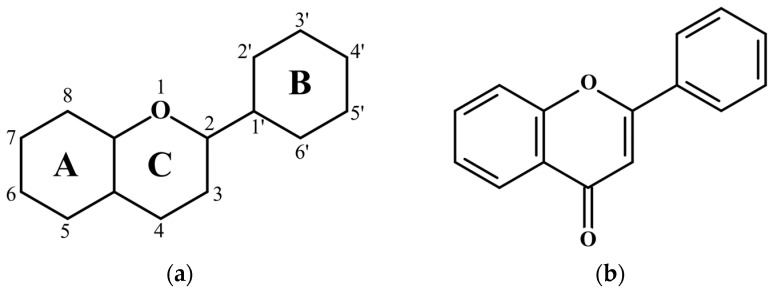
(**a**) Flavonoid backbone; (**b**) Flavone backbone.

**Figure 2:**
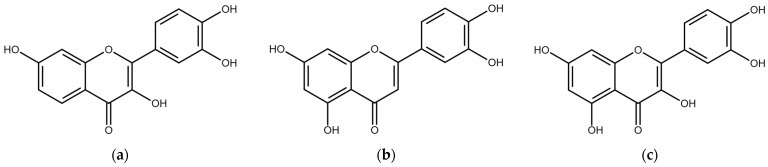
Molecular structure of (**a**) Fisetin; (**b**) Luteolin; (**c**) Quercetin molecules.

The backbone structure of flavonoids, shown in Figure 1a, consists of two benzene rings (A and B), and a pyrane ring (C). All flavonoid molecules can be classified into several subgroups. The backbone of the Flavone subgroup (see Figure 1b) has a carbonyl group attached to the C4 atom and a double bond between the C2 and C3 atoms. The carbonyl group can act as an acceptor for the proton in the ESIPT process if the proton donor groups, such as a hydroxyl (–OH) group, are attached to the neighboring carbon atoms in the A or C rings. The L (3′, 4′, 5, 7–tetrahydroxyflavone) molecule (Figure 2a) has a hydroxyl group in the C5 position, thus allowing the proton H5 transfer towards O4 (H5 ESIPT process). The F (3′, 4′, 3, 7–tetrahydroxyflavone) molecule (Figure 2b) has the H-donor hydroxyl group in the C3 position, and the H3 ESIPT process can occur. The Q (3′, 4′, 3, 5, 7–pentahydroxyflavone) molecule (Figure 2c) has two potential paths for the ESIPT process, as it has both donors neighboring the carbonyl group, and therefore, both ESIPT processes can happen.

Q is the most widely distributed and extensively studied flavonoid [8,26,36,37]. Q powder can be acquired in unhydrated or in different hydrated forms. Earlier, we studied the FL spectra of Q unhydrated [28] and hydrated powders [29]. We also calculated characteristic wavelengths for corresponding molecular structures [38,39]. For this study, we selected another Q powder—Q dihydrate (QDH). Its molecular structure was reported to be the same in two different crystallographic studies [40,41], and in its structure, the –OH3 and –OH5 groups form H-bonds with the O4 atom, thus allowing both ESIPT processes. To the best of our knowledge, neither QDH FL spectra nor correspondent computations have been performed before. The FL spectra of F and L powders and solutions in high concentration have not yet been reported, either.

Thus, F and L molecules have different structures, allowing only one possible proton transfer for each of them: H3 for F and H5 for L. Here, we compare their calculated emission wavelengths with their FL spectra obtained for the corresponding powders and solutions and show that the calculated and experimental results are consistent with the presence of the ESIPT process. We also compare the absorption and emission wavelengths found for F and L with those calculated and measured for Q, where both H3 and H5 transfers are possible.

## 2. Materials and Methods

### 2.1. Experimental Setup

The FL emission was measured using a conventional experimental setup, which included a TRIAX550 monochromator (Horiba/Jobin Yvon) (Kyoto, Japan) and a liquid-nitrogen-cooled charge-coupled-device (CCD) detector. The optical excitation was provided by a laser diode with a wavelength of 405 nm. To avoid degradation, a low excitation light intensity of approximately 5 mW was used in all measurements.

Fisetin (PHL82542 powder with >90% purity), Luteolin (L9283 powder with >98% purity) and Quercetin Dihydrate (337951 powder with >95% purity) were acquired from Sigma-Aldrich (St. Louis, MO, USA). Methanol (>99.5% and >99.9%) was purchased from J. T. Baker (St. Louis, MO, USA). Propylene glycol (≥99.5%) was purchased from Reactivos Quimica Meyer (Mexico City, Mexico).

### 2.2. Computational Details

Density functional theory (DFT) [42,43] with the Minnesota functional M06-2X [44] and 6-31++G(d,p) basis set, implemented in Gaussian 16 [45], was used for geometry optimization and for vibrational frequency calculations. This functional was chosen because it considers the non-planarity of flavonoid molecules in the ground state, which is important to accurately estimate the characteristic energies of the flavonoids FL emission [4]. It has been demonstrated [46] that the M06-2X functional with a state-specific approach shows good results in describing the effect of solvent on the characteristic absorption and emission wavelengths. The time-dependent DFT (TDDFT) [47] approach was used for the geometry optimization and for vibrational frequency calculations in the excited state. The polarizable continuum model (PCM) within the self-consistent reaction field (SCRF) approach [48] was used to consider the influence of the solvents on the absorption and emission energy. For propylene glycol (PG), a generic PCM solvent in Gaussian was used. When modeling the influence of PG, the values of the dielectric constant, ε = 27.50, and refractive squared index, *n*^2^ = 2.052, were used; these parameters were reported in Ref. [49]. The state-specific approach for solvents was used [50]. All calculations were performed using the supercomputer facility of Laboratorio Nacional de Supercomputo del Sureste (LNS), Mexico.

## 3. Results and Discussion

### 3.1. Measured FL Spectra of F, L and QDH Powders and Solutions

**Figure 3:**
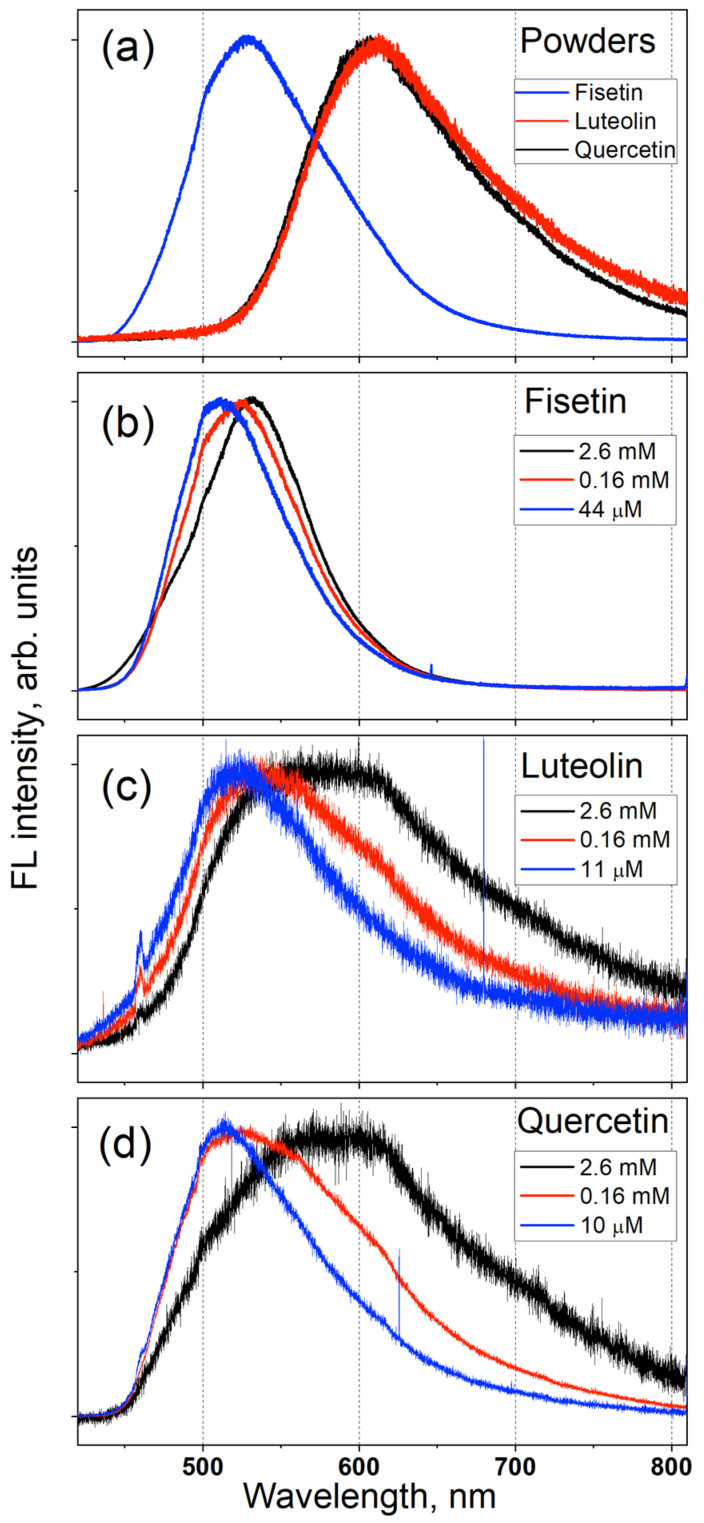
(**a**) FL spectra of F, L and QDH powders. FL spectra of (**b**) F, (**c**) L and (**d**) QDH solutions in methanol with different concentrations. For the sake of clarity, all the spectra were normalized to unity at their maximum value. The sharp peak in (**c**) at approximately 460 nm is due to methanol emission.

In Figure 3a, the measured FL spectra of F, L, and QDH powders are shown. All these FL spectra are broad and have almost the same value for full width at half maximum (FWHM). The spectral positions of their maxima are different and correspond to the wavelengths of 530 nm for F, and of 620 nm for L and QDH.

F, L and QDH are soluble in different types of solvents, as are many other flavonoid powders. Although their solubility in polar solvents is much higher than in non-polar ones, flavonoid powders are known to be poorly soluble in water [51,52,53]. To prepare the solutions, two polar protic solvents were chosen: methanol, because many flavonoids have the highest solubility in this solvent, and propylene glycol, since it is often used in the chemical, food, and pharmaceutical industries.

We have observed earlier that the spectral position of the FL emission peak of some flavonoid powders (Quercetin, Kaempferol, Morin, Myricetin) is time-dependent [28,29,30,31]. This dependence can be explained as follows: undissolved powder clusters still remain in the fresh solution, and it takes a certain time for the flavonoid molecules to separate from each other. This time depends on the solvent, and for methanol, is approximately one to two weeks. The same time-dependent effect after solution preparation was observed for the FL spectra when preparing the F and L solutions, and the time required for complete dissolution of a certain amount of powder was approximately two weeks. Therefore, here, we show the FL spectra of the solutions prepared two weeks before measurement.

In Figure 3b–d, the FL spectra of F, L and QDH solutions in methanol are shown. The spectral position of the FL emission depends on the solution concentration: the spectra of solutions with higher concentrations are similar to those of the corresponding powders, while the spectra of those with lower concentrations are all blue-shifted. The spectral positions of the FL spectral maxima for solutions with low concentrations correspond to approximately 520 nm for F, L and QDH. It is also noticeable that the blue-shift value for F is much smaller than for L and QDH.

**Figure 4:**
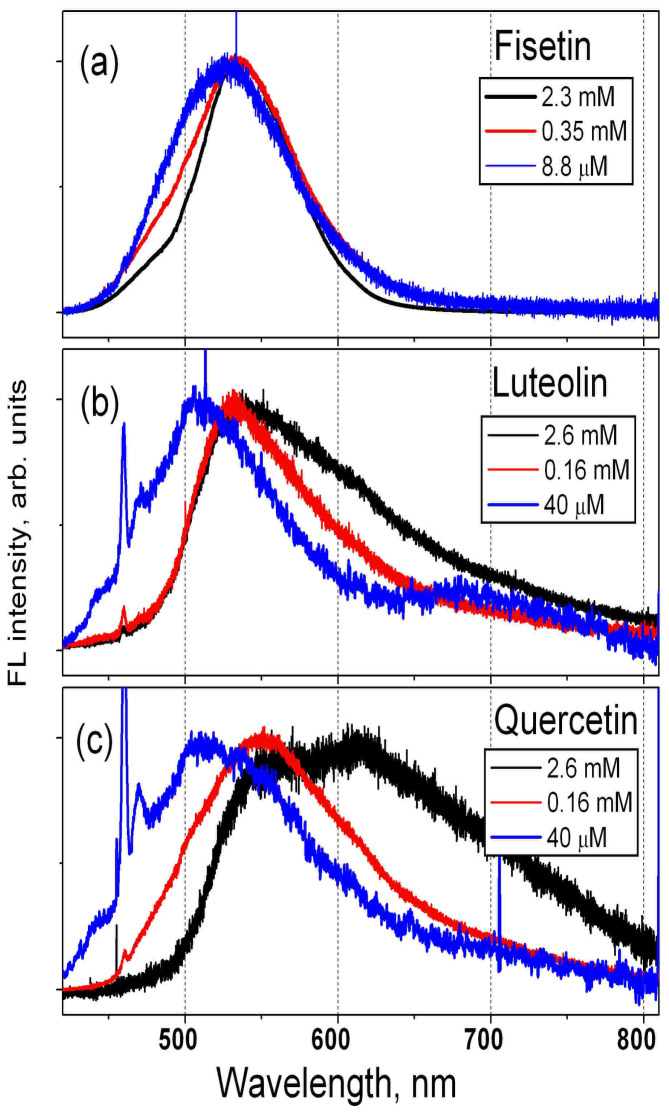
FL spectra of (**a**) F; (**b**) L; (**c**) QDH solutions in propylene glycol with different concentrations. For the sake of clarity, all the spectra were normalized to unity at their maximum value. The sharp peaks at approximately 470 nm are due to propylene glycol emission.

In Figure 4, the FL spectra of F, L and QDH solutions in propylene glycol are shown. The spectral positions of the FL maxima for different concentrations, the values of FWHM, and the dependence of the spectrum shape on the solution concentration are very similar to those obtained for solutions in methanol.

The intensity of the FL emission of F solutions was significantly higher than that of L and QDH solutions and, therefore, it was possible to measure the FL emission from F solutions having lower concentrations.

### 3.2. Calculation of Wavelengths of Absorption and Emission for F, L and Q Molecules

#### 3.2.1. Different Configurations of Flavonoid Molecules: New System of Notation and Selection of the Configuration with the Lowest Potential Energy

Flavonoid molecules usually have several –OH groups, and their computational analysis requires considering various molecular configurations with two different orientations of the H atom in each –OH group. Sometimes, a specific configuration of a molecule can be obtained from the crystal structure of the corresponding flavonoid. When studying the molecular configuration in solvents, it is more appropriate to consider the configuration corresponding to the minimum energy, although a combination of different molecular configurations with similar energies can exist in solutions.

**Figure 5:**
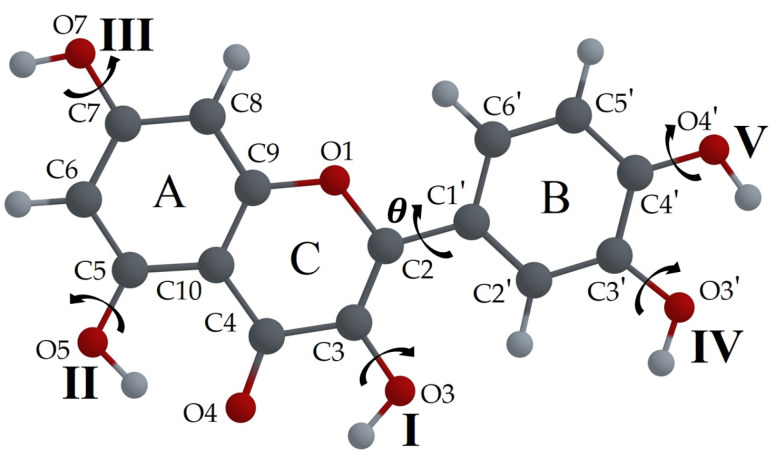
Atom numbering of the Q molecule. Latin numbers indicate the positions of –OH groups in the label. The orientations of hydroxyl groups correspond to the configuration Q00000S; B-ring is in *syn* position.

To distinguish between different molecular configurations, here, we suggest a system of notation based on the molecular structure of Q but also suitable for other flavonoids. The B ring in the Q molecule is asymmetric with respect to the C2-C1′ bond that connects the B and C rings, so, to define the position of the –OH3′ group, the terms “*syn*” and “*anti*” are commonly used. The *syn* orientation corresponds to the case in which the O3 and O3′ atoms are located on the same side of the C2-C1′ bond, as shown in Figure 5, and the *anti* orientation corresponds to the position of these atoms on opposite sides of this bond, as suggested in Ref. [54].

In Figure 5, we use the Latin numbering for hydroxyl groups. We suggest indicating the orientation of each –OH group of the molecule using the sequence QXXXXX, where X values can be equal to “0” or “1” depending on the orientation of the –OH group and their order corresponds to the Latin numbers of the –OH groups. The Q00000 configuration is selected as that with the lowest molecular potential energy of the Q molecule, according to the calculation made using various ab initio and DFT functional methods. Then, the letter “A” is added if the B ring has the orientation *anti*, or “S” for *syn*. For example, the configuration shown in Figure 5 can be labeled as Q00000S. Such notation includes all the information about the specific configuration of the Q molecule.

If a flavonoid molecule has the same number of hydroxyl groups as Q (such as Dihydroquercetin, Catechin or Cyanidin), this system of notation can be used as-is. For flavonoid molecules with a smaller number of –OH groups, such as L and F, one of the digits can be eliminated.

**Figure 6:**
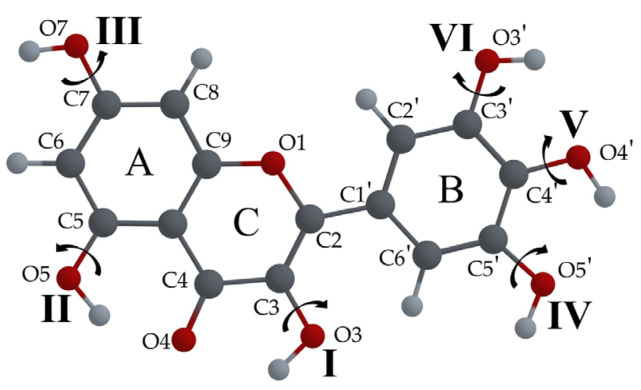
Atom numbering of the Myricetin molecule. Latin numbers indicate the positions of –OH groups in the label. The orientations of hydroxyl groups correspond to the configuration M000000.

For flavonoid molecules such as Myricetin, Gallocatechin or Delphinidin with six –OH groups, there are no *syn* or *anti* distinctions due to the higher degree of symmetry; therefore, to determine each configuration, a six-digit numbering (XXXXXX) scheme can be used. In our previous study [30], we analyzed the configuration of the molecule of Myricetin that corresponds to the minimum energy and, in our notation system, can be labeled as M000000. This configuration of the Myricetin molecule is shown in Figure 6. It can be seen that the orientations of the –OH7, –OH3′ and –OH4′ are the same in Q0000S and M000000 configurations.

**Figure 7:**
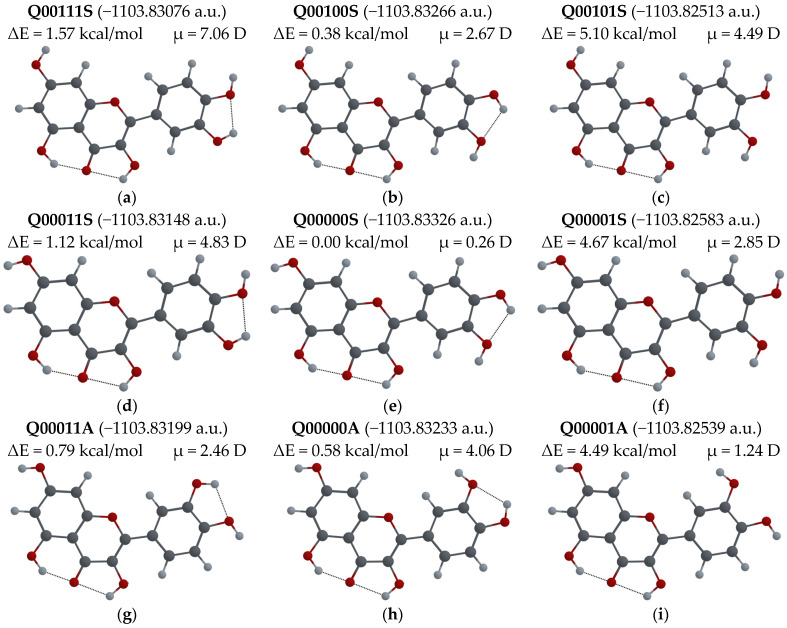

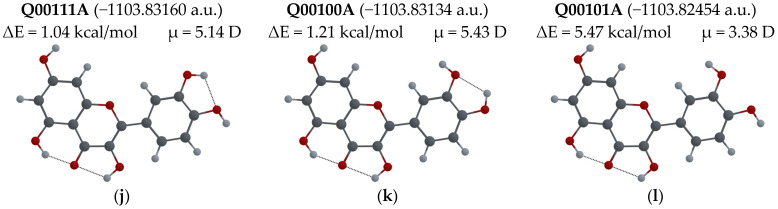
Q molecular configurations: (**a**) Q00111S; (**b**) Q00100S; (**c**) Q00101S; (**d**) Q00011S; (**e**) Q00000S (the lowest energy configuration); (**f**) Q00001S; (**g**) Q00011A; (**h**) Q00000A; (**i**) Q00001A; (**j**) Q00111A; (**k**) Q00100A; (**l**) Q00101A. Total energy, ΔE = E_conf_ − E_min_ energy difference with respect to lowest energy configuration, and μ-dipole moment of the molecule optimized in its ground state are also shown.

**Figure 8:**
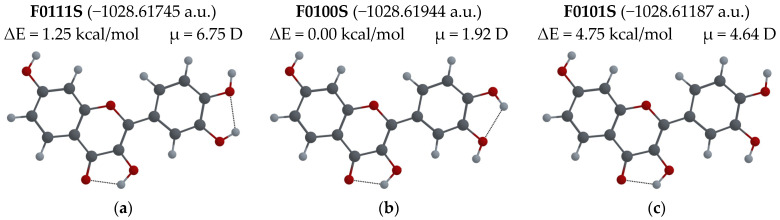

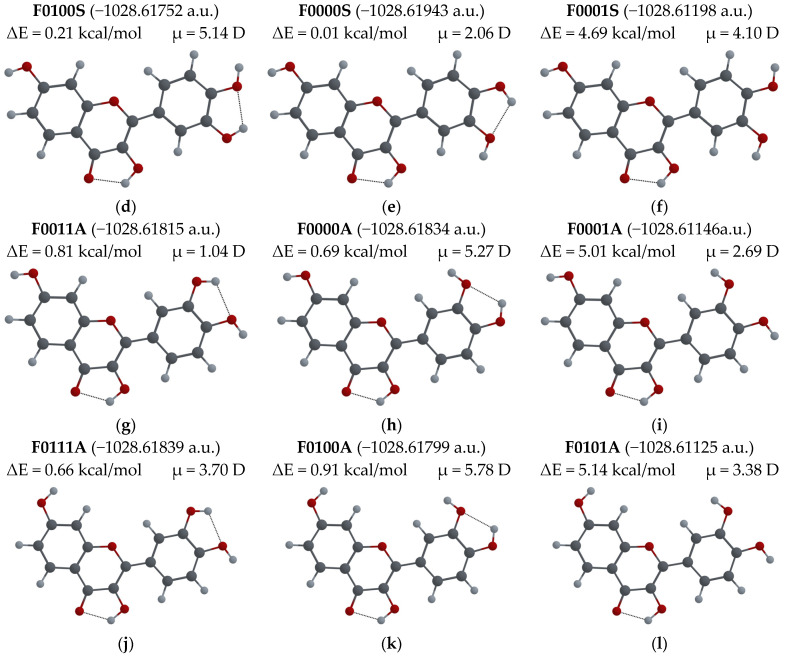
F molecule configurations: (**a**) F0111S; (**b**) F0100S (the lowest energy configuration); (**c**) F0101S; (**d**) F0011S; (**e**) F0000S; (**f**) F0001S; (**g**) F0011A; (**h**) F0000A; (**i**) F0001A; (**j**) F0111A; (**k**) F0100A; (**l**) F0101A. Total energy, ΔE = E_conf_ − E_min_ energy difference with respect to lowest energy configuration, and μ-dipole moment of the molecule optimized in its ground state are also shown.

**Figure 9:**
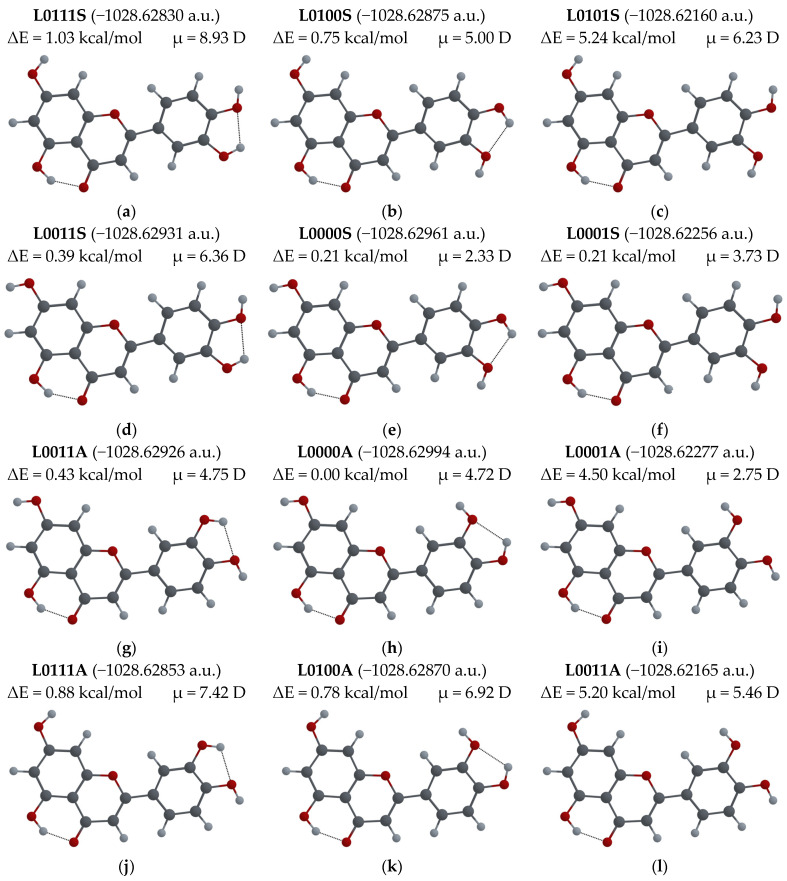
L molecular configurations: (**a**) L0111S; (**b**) L0100S; (**c**) L0101S; (**d**) L0011S; (**e**) L0000S; (**f**) L0001S; (**g**) L0011A; (**h**) L0000A (the lowest energy configuration); (**i**) L0001A; (**j**) L0111A; (**k**) L0100A; (**l**) L0101A. Total energy, ΔE = E_conf_ − E_min_ energy difference with respect to lowest energy configuration, and μ-dipole moment of the molecule optimized in its ground state are also shown.

If two possible orientations are considered for each of the –OH groups that cannot participate in the ESIPT process, 12 different configurations are possible for each of the Q, F and L molecules. For all these molecular configurations, geometry optimization was performed, and the corresponding potential energies were calculated. The obtained values for Q, F and L molecules are shown in Figure 7, Figure 8 and Figure 9, respectively. The Figures also display the difference between the energy of a given configuration and the lowest energy, ΔE = E_conf_ − E_min_ (in kcal/mol), and the dipolar moment μ (in Debye) for each configuration.

In Figure 7, it can be seen that the configuration with the lowest energy, Q00000S, is also the configuration with the lowest dipole moment (0.26 D). The total energy of the Q00111S configuration is only 1.57 kcal/mol greater, although the dipole moment of this configuration changes to 7.06 D. Thus, the value of the dipole moment is very sensitive to changes in the –OH group orientations. Nine configurations of this set were found experimentally by X-ray diffraction in either crystals of quercetin monohydrate [39] (Q00001S), quercetin dihydrate [40,41] (Q00001A), or in cocrystals of quercetin with various substances: tetramethylpyrazine, 4,4′-bipyridine, 1,2-bis(4-pyridyl)ethylene, 1,2-bis(4-pyridyl)ethane, 4,4′-azopyridine and phenazine [55]; caffeine, caffeine with methanol, isonicotinamide and theobromine [53]; and isonicotinic acid [56]. The configurations Q00111A, Q00100A and Q00101A were not observed experimentally, while Q00111S, Q00100S and Q00101S were found in the most common cocrystal structures in [55,57]. Earlier, we reported our results for the calculations of absorption and emission wavelengths for the Q00000S and Q00001S configurations [28,29,30]. Here we will analyze the Q00001A configuration. It was used before in the solid-phase nuclear magnetic resonance studies of QDH powders as a basis for obtaining the configuration of the Q molecule in the crystal structure of unhydrated Q [55,58,59]. The molecular configuration found in that study was Q00011S. However, there is only one experimental structure [38] where the Q unhydrate has been reported, that in our notation corresponds to the Q10100A configuration.

For the F molecule (see Figure 8), the lowest energy configuration is F0100S, and the only difference between this one and the Q00000S configuration is the orientation of the –OH7 group. For the F0000S configuration, which has the same orientation of–OH groups and the same *syn* position of the B ring as the Q00000S, the difference in energy, ΔE, is the smallest, of only 0.01 kcal/mol compared to that of the lowest energy configuration. These configurations have relatively small dipole moments of 1.92 D and 2.06 D, respectively. There are no data on F crystal structure; the only available structure is that of cocrystals with caffeine, F0011A, reported in Ref. [60] This structure has the lowest dipole moment (1.04 D) of all 12 structures, and its energy difference, ΔE, is of 0.81 kcal/mol. In the calculations of absorption and emission wavelengths reported in Ref. [11], the particular molecular configuration was not mentioned, and in Ref. [34], the F0011A configuration was used.

The results of calculations of absorption and emission wavelengths for the enol and keto forms of all F configurations shown in Figure 8 can be found in Table A1 of Appendix A.

For the L molecule (see Figure 9), the calculations show that for the lowest energy configuration, L0000A, the –OH group orientations are the same as those for Q00000S, but the B ring is in the *anti* position. The configuration L0000S, with the same orientations of –OH groups and the same position of the B ring as Q00000S, has the smallest difference in energy ΔE, of 0.21 kcal/mol with respect to the minimum energy configuration. In general, the dipole moments of the L configurations are larger than those of Q and F. For the lowest energy configurations, L0000A and L0000S, μ is 4.72 D and 2.33 D, respectively. The crystal structure of L-hemihydrate is available, and the configuration of the L molecule found there is the L0101A configuration [61]. The L0001A configuration has been found in two cocrystals [62]. The L0100S [13] and L0000A [35] configurations have been used in computational studies. We calculated the absorption and emission wavelengths for the enol and keto forms of 12 different configurations of L molecules, shown in Figure 9. The results can be found in Table A2 in Appendix A.

#### 3.2.2. Tautomeric Forms (Enol and Keto) for F, L and Q Molecules

**Figure 10:**
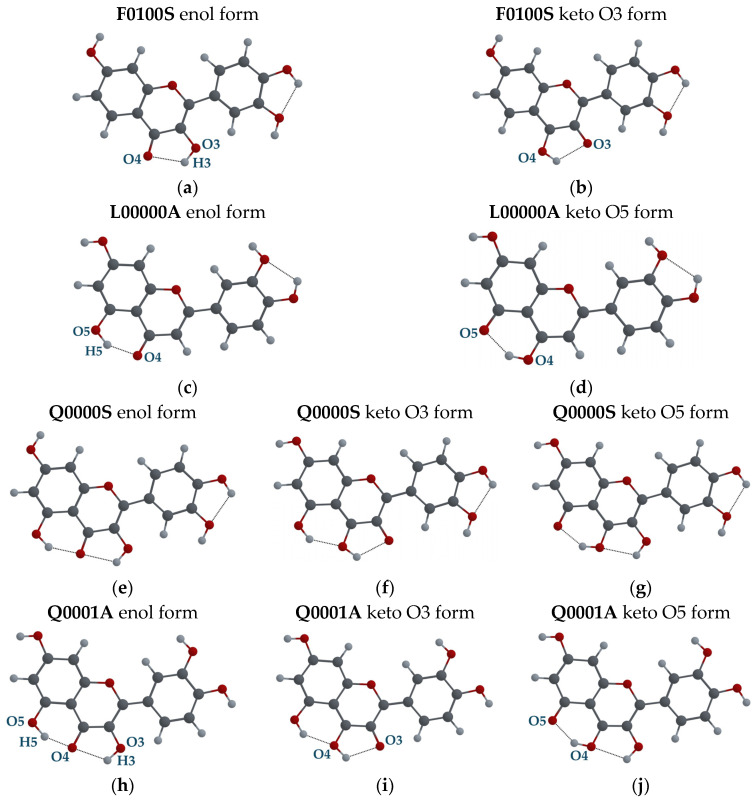
F0100S molecular configuration: (**a**) Enol form; (**b**) Keto O3 form. L0000A molecular configuration: (**c**) Enol form; (**d**) Keto O5 form. Q00000S molecular configuration: (**e**) Enol form; (**f**) Keto O3 form; (**g**) Keto O5 form. Q00001A molecular configuration: (**h**) Enol form; (**i**) Keto O3 form; (**j**) Keto O5 form.

In Figure 10, the possible tautomeric forms of the configurations F0100S, L0000A, Q0000S and Q00001A are shown. The most stable tautomeric forms in the ground state, S_0_, correspond to the enol forms (Figure 10a, Figure 10c, Figure 10e and Figure 10h, respectively). If the H3 atom forms a covalent bond with the O4 atom instead of the O3 atom (see Figure 10b), the F molecule has a tautomeric keto O3 form. The L molecule also has only one keto O5 form when the H5 atom is bonded to the O4 atom (see Figure 10d). The lowest energy configuration, Q0000S, and the configuration Q00001A found in QDH crystal can have keto O3 (see Figure 10f,i) and keto O5 forms (see Figure 10g,j).

#### 3.2.3. Molecular Orbital Analysis

**Figure 11:**
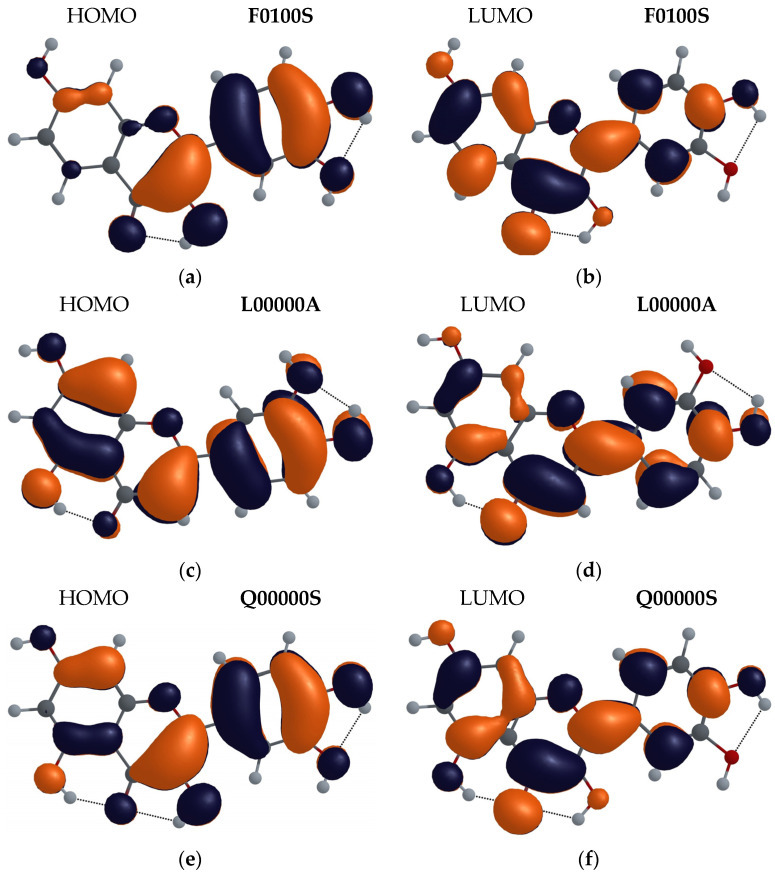
Electron population of F0100S (**a**,**b**), L0000A (**c**,**d**), and Q00000S (**e**,**f**) molecular configurations. (**a**,**c**,**e**) HOMOs and (**b**,**d**,**f**) LUMOs.

For the configurations with the lowest energies of F, L and Q molecules (F0100A, L0000A and Q00000S), we performed an analysis of their frontier molecular orbitals. In Figure 11, the highest occupied molecular orbital (HOMO) and the lowest unoccupied molecular orbital (LUMO) representations of the F, L and Q molecules, obtained by the M06-2X method with the 6-31++G(d,p) basis set, are shown. The HOMO electron population has a π-bonding character and is similarly distributed in the B ring in all three molecules. The L and Q molecules also have similar electron population distributions in the A and C rings. In the O4 of the carbonyl group and in the O3 of the hydroxyl groups of the F and Q molecules, the electron distribution in the HOMO is also similar. In the C ring, the C2-C3 bond has an increased electron population in all three molecules, especially in the F molecule (in which the electron population in the A ring is very low). On the other hand, in the L molecule, the electron population at oxygen O5, along the C5-C10 and C7-C8 bonds in the A ring, is higher than that in the Q molecule.

The electron population in the LUMO of the three molecules is distributed more equally than in the HOMO, and has a π- antibonding character. It decreases on all hydroxyl groups having H-bonds but increases on the O4 carbonyl group and acts for the H5(H3) proton as an additional attraction force towards the O4 atom.

Furthermore, in LUMO, the electron population is transferred from the C2-C3 bond to the C2-C1′ bond that connects the C and B rings, and thus promotes flatter geometries for the L, F and Q molecules in the first excited state. This is consistent with the changes in the angle between C and B rings in the ground state. For the ground state, these angles are: 7.03° for the F molecule, 20.59° for the L molecule, and 6.21° for the Q molecule, while for the first excited state, 0.27° for F enol and almost 0° for the F, L, and Q molecules in their keto forms.

#### 3.2.4. Relaxed Scan of the S_0_ and S_1_ State Energies as a Function of O3-H3 or O5-H5 Distance. Search for the Potential Energy Minima, Corresponding to Enol and Keto Forms

**Figure 12:**
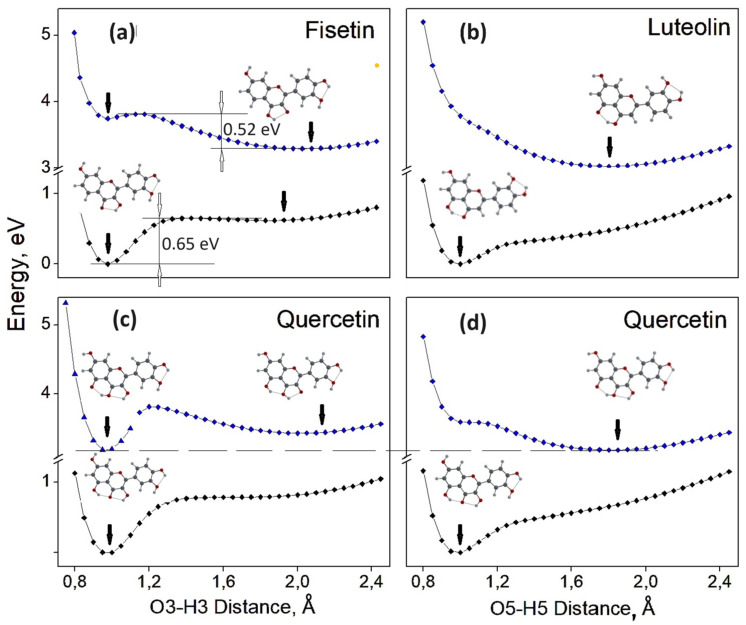
Dependence of the molecule potential energy on the O3-H3 or O5-H5 distance for (**a**) F0100S, (**b**) L0000A, (**c**,**d**) Q00000S molecules. The black curves correspond to the energies in the ground state (S_0_), and the blue curves correspond to the energies in the first excited state (S_1_). Arrows indicate the energy minima.

To better understand the ESIPT process, we performed the relaxed scan of the potential energy for the F0100A, L0000A and Q00000S configurations (having the lowest energy for F, L and Q molecules) as a function of the distance between the oxygen O3 (or O5) and hydrogen atoms of the –OH3 (or –OH5) hydroxyl groups. The resulting curves for the molecular energy in the ground state (black curves) and in the first excited state (blue curves) are shown in Figure 12. Each point on the curve corresponds to the potential energy minimum of the corresponding molecule for each fixed distance between the O5 and H5 (O3 and H3) atoms in the range of 0.8–2.5 Å. For the sake of clarity, we set the zero energy level to the enol minimum in the S_0_ state for all four scans. To obtain more reliable results, we performed each scan in two opposite directions: first from the enol form (which corresponds to short O-H distances) towards the keto form of the molecule, and then back in the opposite direction, from large O-H distances to shorter ones.

For the F0100S configuration in the ground state, S_0_, there are two potential energy minima: at the O3-H3 distance of approximately 0.98 Å, corresponding to the enol form, and at approximately 1.85 Å, corresponding to the keto O3 form (see Figure 12a). There is an energy barrier of 0.65 eV between these two minima, and the energy of the second minimum is 0.62 eV greater than that of the first one. In the first excited state, there are also two energy minima: at the distance of 1 Å and at 2.05 Å, corresponding to the enol and keto O3, respectively. One can see that in the excited state, the minimum of the keto O3 form (corresponding to 3.29 eV) is 0.45 eV lower than that of the enol form, with an energy barrier of 0.52 eV.

The obtained potential energy scans for the L0000A configuration (Figure 12b) are different from those for F0100S: in the S_0_ state, there is only one potential energy minimum, at the O5-H5 distance of approximately 0.99 Å, corresponding to the enol form. In the S_1_ state, there is also only one energy minimum, at the O5-H5 distance of approximately 1.8 Å, corresponding to 3.08 eV, for the keto O5 form. There is no energy minimum for the enol form, and thus, within the L molecule, the ESIPT process can occur spontaneously, since the molecule after photoexcitation cannot have the enol form and changes to the keto O5 tautomeric form.

For the Q00000S configuration of the Q molecule, potential energy curves are shown for two possible intramolecular proton transfers: O3-H3 (Figure 12c) and O5-H5 (Figure 12d). In the S_0_ state, there is only one energy minimum, corresponding to the enol form when the distance between atoms O3-H3 (O5-H5) is 0.98 Å.

The scan for S_0_ of the Q00000S configuration as a function of the O5-H5 distance is very similar to that of the L0000A configuration, and both scans reach their minima at the same distance of approximately 1 Å. For the S_1_ state of the Q00000S and L0000A configurations, the potential energy curves as a function of the O5-H5 distance are also similar: for the Q (L) molecule, the minimum energy of 3.20 eV (3.08 eV) corresponds to the distance of 1.82 Å (1.80 Å). Since the potential curve for Q00000S has an inflection point (at 1.15 Å) rather than a potential barrier, the transfer of the H5 proton can occur spontaneously, as it does in the L molecule.

The scan for S_1_ of the Q00000S configuration as a function of the O3-H3 distance (Figure 12c) has two energy minima: at the distance of 2.0 Å (3.43 eV), which corresponds to the keto O3 form, and at 0.95 Å (3.20 eV), which corresponds to the keto O5 form. For this curve, the energy values in the range from 1.0 Å to 1.4 Å were different for different scan directions, i.e., when the calculation started from the O3-H3 distance of 0.8 Å, and then in the opposite direction, starting at 2.5 Å. It should be considered that when performing a relaxed scan, the position of the H5 proton is not fixed, but changes during the geometry optimization with the change in the position of the H3 proton. This change in the H5 proton position results in different energies for opposite scan directions. As the H3 proton moves from the O3 atom to the O4 atom (over the O3-H3 distance from 0.8 Å to 1.2 Å), the H5 proton also moves toward the O4 atom until the molecule reaches the energy minimum corresponding to the keto O5 form. Thus, the lowest potential energy for the Q00000S molecule after photoexcitation does not correspond to the keto O3 or enol forms, but to the keto O5 form. Moreover, these results are consistent with our data for the Myricetin molecule [30], and probably the same happens in other flavonoid molecules that have two groups: –OH3 and –OH5. In general, the calculation results clearly indicate that in flavonoid molecules containing –OH3 and –OH5 groups, the ESIPT of the H5 atom dominates.

#### 3.2.5. Absorption and Emission Characteristic Wavelengths of L, F and Q Molecules

The absorption and emission wavelengths were calculated for the 12 configurations of F and L molecules shown in Figure 8 and Figure 9. Table A1 and Table A2 (see Appendix A) display corresponding results. In this section, the results of calculations for only some special configurations are shown.

There are no data on the F molecule configuration in the crystal structure; therefore, to calculate the characteristic absorption and emission wavelengths, we chose the two lowest energy configurations, F0100S and F0000S, which differ in energy by only 0.32 meV (0.01 kcal/mol). For the L molecule, the lowest energy configuration, L0000A, and the configuration reported in crystals [61], L0101A, were chosen. The selected configuration of the Q molecule, Q00001A, was that reported in Q dihydrate crystals [40,41]. The reason for choosing this particular configuration was discussed in the Section 1.

Table 1 shows the results of calculations in vacuum for the configurations F0100S, F0000S, L0000A, L0101A and Q00001A in keto and enol forms. The characteristic wavelengths for the lowest energy configuration, Q0000S, were calculated in our previous study [28]. Table 1 contains the values corresponding to the molecular energies for the ground state E_S0_ (E_S0_*) and for the first excited state E_S1_ (E_S1_*) for the optimized geometry for the ground state (optimized geometry for the first excited state). The calculated absorption (E_S1_ − E_S0_) and emission (E_S1_* − E_S0_ *) energies and the corresponding characteristic wavelengths (λ_ab_ and λ_em_) are shown. The oscillator strengths (f_ab_, f_em_) and dipole moments (μ_S0_, μ_S1_) in the ground state and in the first excited state corresponding to the optimized molecular geometries are also presented. Where the data are absent, the corresponding energy minimum was not found.

All the calculated emission wavelengths corresponding to F keto O3, L keto O5 and Q keto O5 are in very good agreement with the spectral positions of the maxima of the FL spectra measured in powders. The calculated emission wavelength for the F keto O3 form in vacuum is approximately 490 nm. The wavelength values obtained for the 12 configurations of the F molecule are between 489 and 499 nm (see Table A1 of Appendix A). The maxima of the FL spectra of the F powder and F solutions with high concentration were found at approximately 530 nm (see Figure 3a,b and Figure 4a). The calculated emission wavelengths of the keto O5 forms of the L0101A and L0000A configurations (623 nm and 603 nm, respectively) are very close to the spectral position of the maximum of the FL spectrum of the L powder at 620 nm. The wavelength values obtained for the 12 configurations of the L molecule are between 603 and 631 nm (see Table A2 of Appendix A). The calculated emission wavelength of the keto O5 form of configuration Q00001A (591 nm) is close to the spectral position of the maximum of the FL spectrum of QDH powder (620 nm), while the FL emission band corresponding to the keto O3 form of the Q00001A configuration was absent in the FL spectra of the QDH powder. It is worth mentioning that in our previous study [30] of the FL emissions of Kaempferol and Myricetin powders (Kaempferol and Myricetin molecules also have OH groups in the 3 and 5 positions), the emission band related with the keto O3 form was not found either. Thus, our experimental data suggest that under photoexcitation of flavonoid molecules, the O5 keto tautomer form is more likely to be formed than the O3 keto tautomer. This is consistent with the results obtained for the potential energy curves of the Q molecule, which indicate that the ESIPT of the H5 atom dominates. The emissions of the F and Q molecule enol forms, at 355 nm and of 371 nm, respectively, cannot be observed when using an excitation wavelength of 405 nm.

In Table 2, the results of calculations for the same configurations of F, L and Q molecules in the solvents, methanol and propylene glycol, are shown.

The calculation results for solutions of F, L and Q in PG are very similar to the results obtained for solutions in methanol. The values found for F molecule for both solvents are close to those obtained in vacuum. The emission wavelength calculated for solvents is approximately 475 nm; this is 15 nm lower than that calculated for vacuum, and it is consistent with the spectral position of the maximum of the FL spectra of F solutions with low concentration, which is at approximately 515 nm (see Figure 3b and Figure 4a). For the solution of L in methanol, the emission wavelength value for the keto form of the L0000A configuration (L0101A) was 544 nm (580 nm). The maximum of the FL spectrum of the L solution in methanol with low concentration was found at 525 nm, i.e., closer to the value obtained for the minimum energy configuration (L0000A). For the Q solutions, the calculated emission wavelength for the keto form O3 (O5) was 481 nm (530 nm), and these wavelengths are close to the value of 515 nm that corresponds to the maximum of the FL spectrum of the QDH solution in methanol with low concentration. The emission wavelength found in our previous study [28] for the keto O5 form of the lowest energy configuration of the Q molecule was 551 nm. As the concentration of the solution increases, the FL spectrum shifts to longer wavelengths, and the FL spectra of solutions with high concentrations become closer to the spectra of the powder. We observed and analyzed a similar dependence of the FL spectra on solution concentration in our early studies of several flavonoids [28,29,30].

## 4. Conclusions

- To better show the difference between the ESIPT processes involving different protons, F and L molecules were chosen, since only one proton can be transferred in each of them. We also analyzed the ESIPT in the Q molecule, where the transfer for two protons is possible.
- To easily distinguish between different configurations of flavonoid molecules, a new system of notation for all possible configurations of the molecules was developed.
- For several possible configurations of F, L and Q molecules, a search for the configuration with lowest potential energy was performed.
- Analysis of the electron populations of frontier molecular orbitals, HOMO and LUMO, performed for the lowest energy configurations of F, L and Q molecules, indicated that for all three molecules, the keto forms are favored in the first excited state.
- Relaxed scans of potential energy as a function of the distance between the corresponding O and H atoms were performed for the F, L, and Q molecules. It was found that in the Q molecule, the H5 proton transfer dominates, which is probably common for all flavonoid molecules that have both H3 and H5 protons.
- For the lowest energy configurations of the F, L and Q molecules, the characteristic wavelengths of absorption and emission were calculated using density functional theory (DFT), and the polarizable continuum model (PCM) was used to consider the influence of the solvents on the absorption and emission energy.
- The FL spectra of F, L and Q powders and their solutions in methanol and propylene glycol were measured. The calculation results for vacuum agreed well with the experimental FL spectra of powders and solutions with high concentration; and the calculation results for solutions, with those measured for solutions with low concentrations.
- The fact that the differences in the spectral positions of the maxima of the measured FL spectra of F and L powders correspond to the calculated emission wavelengths confirms the effectiveness of the ESIPT model used for the calculations and the accuracy of its predictions.

## Figures and Tables

**Table 1:** Absorption and emission wavelengths for enol and keto forms of selected configurations of F, L and Q molecules obtained at TDDFT-M06-2X/6-31++G(d,p) level of theory.

	E_S0_ (a.u)	E_S1_ (a.u)	E_S1_ − E_S0_ (eV)	λ_ab_ (nm)	f_ab_	μ_S0_ (D)	E_S1_* (a.u)	E_S0_* (a.u)	E_S1_* − E_S0_* (eV)	λ_em_ (nm)	f_em_	μ_S1_ (D)
	S_0_ Opt	S_0_ Opt					S_1_ Opt	S_1_ Opt				
**Fisetin**												
F0100S enol	−1028.61944	−1028.47277	3.9911	**310.65**	0.6232	1.92	−1028.48200	−1028.61049	3.4962	**354.63**	0.6733	2.35
F0100S keto	−1028.59672	−1028.49262	2.8325	**437.72**	0.5277	3.68	−1028.49846	−1028.59158	2.5341	**489.28**	0.5148	3.15
F0000S enol	−1028.61943	−1028.47404	3.9563	**313.38**	0.6245	2.06	−1028.48303	−1028.61063	3.4722	**357.08**	0.6709	1.92
F0000S keto	−1028.59616	−1028.49317	2.8025	**442.41**	0.5292	4.80	−1028.49880	−1028.59120	2.5145	**493.08**	0.5154	4.31
**Luteolin**												
L0000A enol	−1028.62994	−1028.47479	4.2217	**293.68**	0.4830	4.72	-	-	-	-	-	-
L0000A keto	-	-	-	-	-	-	−1028.51678	−1028.59237	2.0570	**602.75**	0.0541	7.06
L0101A enol	−1028.62165	−1028.46553	4.2484	**291.84**	0.3954	5.45	-	-	-	-	-	-
L0101A keto	-	-	-	-	-	-	−1028.50814	−1028.58125	1.9895	**623.19**	0.0399	9.77
**Quercetin**												
Q00001A enol	−1103.82539	−1103.68525	3.8134	**325.17**	0.6137	1.24	−1103.69358	−1103.81652	3.3470	**370.48**	0.6121	1.77
Q00001A keto O3	-	-	-	-	-	-	−1103.69792	−1103.78879	2.4728	**501.46**	0.5086	3.32
Q00001A keto O5	-	-	-	-	-	-	−1103.70799	−1103.78516	2.0999	**590.50**	0.1009	5.12

Note: **E_S0_** denotes the energy of the molecule when optimized in its ground state (**S_0_**); E_S1_* is the energy of the first excited state (**S_1_**) at the geometry optimized for this excited state; E_S0_* indicates the energy of the ground state (**S_0_**) at the geometry optimized for the first excited state (**S_1_**); **E_S1_** is the energy of the first excited state (**S_1_**) at the ground state optimized geometry (**S_0_**); **f_em_** and **f_ab_** are the oscillator strengths for emission and absorption, respectively; **μ**_S0_ and **μ**_S1_ are the dipole moments in the ground state and first excited state optimized geometries, respectively.

**Table 2:** Absorption and emission wavelengths for enol and keto forms of selected configurations of F, L and Q molecules in implicit solvents methanol and propylene glycol, obtained at PCM/TDDFT-M06-2X/6-31+G(d,p) level of theory.

	E_S0_ (a.u)	E_S1_ (a.u)	E_S1_ − E_S0_ (eV)	λ_ab_ (nm)	f_ab_	μ_S0_ (D)	E_S1_* (a.u)	E_S0_* (a.u)	E_S1_* − E_S0_* (eV)	λ_em_ (nm)	f_em_	μ_S1_ (D)
	S_0_ Opt	S_0_ Opt					S_1_ Opt	S_1_ Opt				
**METHANOL**												
**Fisetin**												
F0100S enol	−1028.63734	−1028.49589	3.8492	**322.11**	0.7242	3.15	−1028.50771	−1028.62438	3.1745	**390.56**	1.0327	4.12
F0100S keto	−1028.61643	−1028.50454	3.0445	**407.24**	0.6319	5.55	−1028.51289	−1028.60894	2.6135	**474.40**	0.8204	4.62
F0000S enol	−1028.63717	−1028.49566	3.8508	**321.97**	0.724	2.76	−1028.50759	−1028.62445	3.1800	**389.89**	1.0336	2.01
F0000S keto	−1028.61600	−1028.50446	3.0351	**408.50**	0.6325	6.99	−1028.51285	−1028.60862	2.6062	**475.73**	0.8202	5.97
**Luteolin**												
L0000A enol	−1028.64902	−1028.50110	4.0252	**308.02**	0.0003	6.67	-	-	-	-	-	-
L0000A keto	-	-	-	-	-	-	−1028.5402	−1028.624	2.2788	**544.08**	0.3191	8.77
L0101A enol	−1028.64580	−1028.49820	4.0164	**308.70**	0.6991	7.51	-	-	-	-	-	-
L0101A keto	-	-	-	-	-	-	−1028.5356	−1028.6142	2.1388	**579.69**	0.3000	12.03
**Quercetin**												
Q00001A enol	−1103.84661	−1103.70812	3.7684	**329.02**	0.7071	2.03	−1103.71784	−1103.83612	3.2185	**385.23**	1.0064	3.11
Q00001A keto O3	−1103.81936	−1103.70920	2.9975	**413.63**	0.6337	5.75	−1103.71748	−1103.81215	2.5761	**481.29**	0.7960	5.05
Q00001A keto O5	-	-	-	-	-	-	−1103.73257	−1103.81851	2.3384	**530.22**	0.4758	5.75
**PROPYLENE GLYCOL**												
**Fisetin**												
F0100S enol	−1028.63716	−1028.50829	3.5069	**353.54**	0.7478	3.14	−1028.50746	−1028.62550	3.2121	**385.99**	1.0294	4.11
F0100S keto	−1028.61623	−1028.50724	2.9657	**418.06**	0.651	5.53	−1028.51275	−1028.60949	2.632	**471.07**	0.8177	4.61
F0000S enol	−1028.63699	−1028.50793	3.5120	**353.03**	0.7475	2.75	−1028.50734	−1028.62556	3.217	**385.41**	1.0302	2.01
F0000S keto	−1028.61580	−1028.50714	2.9568	**419.32**	0.6514	6.97	−1028.51271	−1028.60915	2.6244	**472.44**	0.8176	5.95
**Luteolin**												
L0000A enol	−1028.64884	−1028.51396	3.6700	**337.83**	0.7272	6.65	-	-	-	-	-	-
L0000A keto	-	-	-	-	-	-	−1028.54001	−1028.62368	2.2767	**544.58**	0.3142	8.76
L0101A enol	−1028.64502	−1028.51140	3.6360	**341.00**	0.7481	7.51	−1028.51463	−1028.63366	3.2389	**382.80**	1.0091	10.28
L0101A keto	-	-	-	-	-	-	−1028.53530	−1028.61932	2.2863	**542.30**	0.0688	12.03
**Quercetin**												
Q00001A enol	−1103.84650	−1103.71263	3.6426	**340.38**	0.7296	2.0305	−1103.71760	−1103.83593	3.2198	**385.07**	1.0030	3.10
Q00001A keto O3	−1103.81910	−1103.71084	2.9459	**420.88**	0.6532	5.7260	−1103.71727	−1103.81243	2.5892	**478.85**	0.7938	5.02
Q00001A keto O5	-	-	-	-	-	-	−1103.73235	−1103.81821	2.3365	**530.64**	0.4704	5.74

Note: **E_S0_** denotes the energy of the molecule when optimized in its ground state (**S_0_**); E_S1_* is the energy of the first excited state (**S_1_**) at the geometry optimized for the first excited state (**S_1_**), obtained from the equilibrium solvation state-specific calculation; E_S0_* indicates the energy of the ground state (**S_0_**) with nonequilibrium solvation at the geometry optimized for the first excited state (**S_1_**); **E_S1_** is the energy of the first excited state (**S_1_**) at the ground state optimized geometry (**S_0_**) obtained from the nonequilibrium solvation state-specific calculation; **f_em_** and **f_ab_** are the oscillator strengths for emission and absorption, respectively; **μ**_S0_ and **μ**_S1_ are the dipole moments in the ground state and first excited state optimized geometries, respectively.

## Data Availability

Data are contained within the article and can also be obtained from the corresponding authors.

## Appendix A

Table A1 and Table A2 display the absorption and emission wavelengths for the 12 molecular configurations of F and L shown in Figure 8 and Figure 9. For the F molecule, the absorption (emission) wavelengths for the enol form range from 305.87 to 315.75 nm (from 351.67 to 359.13 nm), and for the keto form, from 435.14 to 447.28 (from 488.75 to 499.08 nm). For the L molecule, the absorption wavelengths of the enol form range from 289.28 to 294.02 nm, and the emission wavelengths of the keto form range from 602.75 to 631.41 nm.

**Table A1:** Absorption and emission wavelengths for keto and enol forms of 12 F molecule configurations obtained at TDDFT-M06-2X/6-31++G(d,p) level of theory.

	E_S0_ (a.u)	E_S1_ (a.u)	E_S1_ − E_S0_ (eV)	λ_ab_ (nm)	f_ab_	μ_S0_ (D)	E_S1_* (a.u)	E_S0_* (a.u)	E_S1_* − E_S0_* (eV)	λ_em_ (nm)	f_em_	μ_S1_ (D)
	S_0_ Opt	S_0_ Opt					S_1_ Opt	S_1_ Opt				
F0000A enol	−1028.61834	−1028.47404	3.9267	**315.75**	0.6253	5.2699	−1028.48288	−1028.60976	3.4524	**359.13**	0.6719	5.6120
F0000A keto	−1028.59333	−1028.49146	2.7720	**447.28**	0.5319	7.5186	−1028.49710	−1028.58840	2.4843	**499.08**	0.5110	7.1521
F0000S enol	−1028.61943	−1028.47404	3.9563	**313.38**	0.6245	2.0566	−1028.48303	−1028.61063	3.4722	**357.08**	0.6709	1.9217
F0000S keto	−1028.59616	−1028.49317	2.8025	**442.41**	0.5292	4.8021	−1028.49880	−1028.59120	2.5145	**493.08**	0.5154	4.3103
F0011A enol	−1028.61815	−1028.47136	3.9943	**310.41**	0.5901	1.0451	−1028.48044	−1028.60909	3.5006	**354.19**	0.6381	1.4394
F0011A keto	−1028.59368	−1028.49008	2.8189	**439.84**	0.5104	2.6407	−1028.49604	−1028.58849	2.5159	**492.81**	0.4889	2.3030
F0011S enol	−1028.61752	−1028.47003	4.0133	**308.94**	0.5136	5.1418	−1028.47969	−1028.60824	3.4979	**354.46**	0.6264	5.6228
F0011S keto	−1028.59204	−1028.48909	2.8014	**442.58**	0.4885	6.7982	−1028.49523	−1028.58680	2.4918	**497.58**	0.4724	6.5741
F0100S enol	−1028.61944	−1028.47277	3.9911	**310.65**	0.6232	1.9192	−1028.48200	−1028.61049	3.4962	**354.63**	0.6733	2.3551
F0100S keto	−1028.59672	−1028.49262	2.8325	**437.72**	0.5277	3.6813	−1028.49846	−1028.59158	2.5341	**489.28**	0.5148	3.1464
F0100A enol	−1028.61799	−1028.47226	3.9656	**312.65**	0.6195	5.7780	−1028.48149	−1028.60922	3.4756	**356.73**	0.6750	6.2600
F0100A keto	−1028.59346	−1028.49050	2.8017	**442.53**	0.5306	7.4229	−1028.49633	−1028.58836	2.5041	**495.12**	0.5114	7.0050
F0111A enol	−1028.61839	−1028.47031	4.0296	**307.69**	0.5893	3.6960	−1028.47962	−1028.60918	3.5256	**351.67**	0.6422	4.0375
F0111A keto	−1028.59443	−1028.48972	2.8493	**435.14**	0.5096	3.0845	−1028.49586	−1028.58908	2.5368	**488.75**	0.4896	2.8868
F0111S enol	−1028.61745	−1028.46848	4.0535	**305.87**	0.5692	6.7527	−1028.47848	−1028.60794	3.5228	**351.95**	0.6293	7.2079
F0111S keto	−1028.59245	−1028.48836	2.8327	**437.70**	0.4871	7.5223	−1028.49470	−1028.58702	2.5122	**493.54**	0.4716	7.2479
F0001A enol	−1028.61146	−1028.46703	3.9301	**315.47**	0.6180	2.6925	−1028.47576	−1028.60298	3.4618	**358.16**	0.6692	2.8792
F0001A keto	−1028.58676	−1028.48446	2.7836	**445.41**	0.5344	5.2281	−1028.49013	−1028.58186	2.4960	**496.73**	0.5131	4.8196
F0001S enol	−1028.61198	−1028.46658	3.9564	**313.38**	0.6118	4.1028	−1028.47554	−1028.60322	3.4744	**356.86**	0.6616	4.3161
F0001S keto	−1028.58819	−1028.48541	2.7968	**443.31**	0.5191	6.5756	−1028.49112	−1028.58320	2.5056	**494.82**	0.5062	6.1663
F0101A enol	−1028.61125	−1028.46539	3.9689	**312.39**	0.6129	3.0937	−1028.47451	−1028.60259	3.4854	**355.72**	0.6725	3.5565
F0101A keto	−1028.58703	−1028.48364	2.8133	**440.72**	0.5333	4.6620	−1028.48950	−1028.58198	2.5164	**492.70**	0.5139	4.1789
F0101S enol	−1028.61187	−1028.46512	3.9933	**310.48**	0.6083	4.6401	−1028.47438	−1028.60295	3.4987	**354.37**	0.6635	5.0297
F0101S keto	−1028.58864	−1028.48474	2.8272	**438.54**	0.5167	6.3011	−1028.49067	−1028.58347	2.5253	**490.98**	0.5052	5.8538

**Table A2:** Absorption and emission wavelengths for 12 L molecule configurations obtained at TDDFT-M06-2X/6-31++G(d,p) level of theory.

	E_S0_ (a.u)	E_S1_ (a.u)	E_S1_ − E_S0_ (eV)	λ_ab_ (nm)	f_ab_	μ_S0_ (D)	E_S1_* (a.u)	E_S0_* (a.u)	E_S1_* − E_S0_* (eV)	λ_em_ (nm)	f_em_	μ_S1_ (D)
	S_0_ Opt	S_0_ Opt					S_1_ Opt	S_1_ Opt				
L0000A enol	−1028.62994	−1028.47479	4.2217	**293.68**	0.4830	4.7157	-	-	-	-	-	-
L0000A keto	-	-	-	-	-	-	−1028.51678	−1028.59237	2.0570	**602.7**	0.0541	7.0613
L0000S enol	−1028.62961	−1028.47377	4.2405	**292.38**	0.4699	2.3281	-	-	-	-	-	-
L0000S keto	-	-	-	-	-	-	−1028.51661	−1028.59140	2.0351	**609.2**	0.0484	7.3474
L0011A enol	−1028.62926	−1028.47299	4.2522	**291.58**	0.3734	4.7458	-	-	-	-	-	-
L0011A keto	-	-	-	-	-	-	−1028.51660	−1028.59104	2.0256	**612.0**	0.3734	9.6384
L0011S enol	−1028.62931	−1028.47206	4.2790	**289.76**	0.3408	6.3643	-	-	-	-	-	-
L0011S keto	-	-	-	-	-	-	−1028.51635	−1028.59135	2.0407	**607.5**	0.0479	9.4537
L0100S enol	−1028.62875	−1028.47180	4.2706	**290.32**	0.4091	5.0034	-	-	-	-	-	-
L0100S keto	-	-	-	-	-	-	−1028.51536	−1028.58785	1.9727	**628.5**	0.0360	9.8519
L0100A enol	−1028.62870	−1028.47239	4.2534	**291.50**	0.3954	6.9163	-	-	-	-	-	-
L0100A keto	-	-	-	-	-	-	−1028.51520	−1028.58851	1.9948	**621.5**	0.0406	9.9094
L0111A enol	−1028.62853	−1028.47150	4.2730	**290.16**	0.0011	7.4161	-	-	-	-	-	-
L0111A keto	-	-	-	-	-	-	−1028.51556	−1028.58772	1.9636	**631.4**	0.0362	12.1958
L0111S enol	−1028.62830	−1028.47079	4.2859	**289.28**	0.0527	8.9325	-	-	-	-	-	-
L0111S keto	-	-	-	-	-	-	−1028.51500	−1028.58776	1.9798	**626.2**	0.0360	12.3102
L0001A enol	−1028.62277	−1028.46780	4.2169	**294.02**	0.4742	2.7508	-	-	-	-	-	-
L0001A keto	-	-	-	-	-	-	−1028.50962	−1028.58504	2.0522	**604.1**	0.0532	7.0977
L0001S enol	−1028.62256	−1028.46659	4.2443	**292.12**	0.4753	3.7317	-	-	-	-	-	-
L0001S keto	-	-	-	-	-	-	−1028.50938	−1028.58440	2.0415	**607.3**	0.0364	7.0608
L0101A enol	−1028.62165	−1028.46553	4.2484	**291.84**	0.3954	5.4552	-	-	-	-	-	-
L0101A keto	-	-	-	-	-	-	−1028.50814	−1028.58125	1.9895	**623.1**	0.0399	9.7710
L0101S enol	−1028.62160	−1028.46454	4.2738	**290.11**	0.3947	6.2317	-	-	-	-	-	-
L0101S keto	-	-	-	-	-	-	−1028.50806	−1028.58073	1.9775	**626.9**	0.0364	9.8860
